# Induced pH-dependent shift by local surface plasmon resonance in functionalized gold nanorods

**DOI:** 10.1186/1556-276X-8-103

**Published:** 2013-02-22

**Authors:** Yon-Rui Toh, Pyng Yu, Xiaoming Wen, Jau Tang, Tao-shih Hsieh

**Affiliations:** 1 Research Center for Applied Sciences, Academia Sinica, Taipei, Taiwan; 2 Institute of Cellular and Organismic Biology, Academia Sinica, Taipei, Taiwan

**Keywords:** Gold nanorods, pH sensor, LSPR, 11-mercaptoundecanoic acid

## Abstract

Localized surface plasmon resonance (LSPR) spectroscopy of metallic nanoparticles is a powerful tool for chemical and biological sensing experiments. In this study, we observed LSPR shifts of 11-mercaptoundecanoic acid modified gold nanorods (GNR-MUA) for the pH range of 6.41 to 8.88. We proposed a mechanism involving changes of the dipole moment after protonation/deprotonation carboxylic groups of 11-mercaptoundecanoic acid (MUA) which plays an important role by modulating LSPR around the functionalized GNR. Such a stable and easily prepared GNR-MUA has potential to become one of the most efficient and promising pH nanosensors to study intra- or extra-cellular pH in a wide range of chemical or biological systems.

## Background

Gold nanoparticles have gained tremendous attention in recent years due to their attractive properties ranging from easy chemical synthesis, high biocompatibility, particular optical properties characterized by shape- and size-dependent localized surface plasmon resonance (LSPR), and locally enhanced-electromagnetic field. Among these noble metal plasmonic nanoparticles, gold nanorods (GNR) in particular, with its varied size, low reactivity, unique anisotropy shape, and optical properties, have been widely investigated by many research groups [1-3]. On the other hand, the LSPR frequency shifting has been widely used in chemical, gas [4] and bio-sensors [5], to examine the chirality of molecules [6] and be used as an electromagnetic energy transmitter [7] based on various types of pure- [8] or modified-metallic nanostructure array on glass substrate or nanoparticles in bulk solution [9]. In fact, developing of nanoparticle-based sensing materials is important and urgent for detection in special environment, for example, detection of single molecule analyte of internal cell [10-12]. The free-label or monolayer/functionalized nanosensors have been achieved by fluorescence protein [13,14], polymer [15,16], quantum dots (QDs) [17], graphene oxide [18], and metal nanoparticles [19] through monitoring the variations in their fluorescence intensity or lifetime. However, the intrinsic drawbacks of fluorescence probe are photo-bleaching and blinking [20]. Furthermore, the cytotoxicity of the QDs makes them practically useless for *in vivo* biological application. Therefore, it is an urgent task to develop biocompatible and highly photostable nanoparticles for nanosensors, in particular, based on the extinction/scattering, and therefore, with non-blinking is highly preferential. Recently, Zijlstra et al. have demonstrated a label-free optical detection of single non-absorbing molecules by monitoring the plasmon resonance of nanorod via a sensitive photothermal spectra [21]. Generally speaking, optical sensors of metallic nanoparticles can be achieved by exploiting the sensitivity to local refractive index (*n*) of the surrounding medium (Δ*λ*_max_ ≈ Δ*n*) or to the plasmon band shift that is caused by the proximity of nanoparticles [21-24].

In this study, we investigate the pH-dependent local surface plasmon shift in a functionalized GNR. The gold nanorods modified by 11-mercaptoundecanoic acid (GNR-MUA) exhibit excellent stability and are easy to prepare, therefore can be the outstanding potential candidate for nanosensors. More importantly, it is based on the extinction spectrum (scattering) and thus non-blinking. We verified this optical signal originates neither from the aggregation of nanorods nor the variation of refractivity index through ion strength test and the pH titration procedure by comparing a modified pH-independent molecule (1-undecanethiol (UDT)) with MUA. We speculate that the dipole moment changes of MUA ligands on a rod surface play a very important role in this nanoparticle based-sensing system.

## Methods

### Materials

The materials used for the synthesis, hydrogen tetrachloroaurate (HAuCl_4_), sodium borohydrate (NaBH_4_), cetyltrimethylammonium bromide (CTAB), silver nitrate (AgNO_3_), l-ascorbic and ethanol, 95% (EtOH), nitric acid (HNO_3_), and sodium hydroxide (NaOH) 1 M were purchased from Sigma-Aldrich Corporation (St. Louis, MO, USA). 11-Mercaptopropionic acid (MUA) and UDT were of analytical grade and were obtained from Fluka (New South Wales, Australia). All standard chemical solutions or powders were protected from sunlight and kept at 25°C in a well-ventilated chemical storage cabinet and dry box. Stock solutions of sodium borohydride and l-ascorbic acid were freshly prepared for each new set of experiments.

### Synthesis and sample fabrication

The GNRs (4.23 M) used in this study were synthesized by using the seed-mediated growth method in the presence of silver ions [25]. A 0.01 M MUA solution was prepared by mixing 0.04 g of MUA with 19.96 mL ethanol. A same concentration of UDT solution with MUA was prepared as mentioned above. The as-synthesized GNR was washed and centrifuged (6,000 rpm, 6 min) before 100 μL of MUA/UDT was added (remove excess cetyltrimethylammonium bromide (CTAB) surfactant). The LSPR peak of the samples was remained constant after 3 h of reaction time. Finally, the modified samples were washed before use to avoid unpredictable interferences from the free carboxylic groups of MUA in solutions.

### Spectroscopic measurements

The morphology of each specimen was verified through TEM analysis (JEOL, JEM-1200EX 2, Akishima, Tokyo, Japan) operating at 80 kV. A double-beam UV–vis spectrophotometer (JASCO V-670, Easton, MD, USA) with a light path of 10 mm was used to measure the surface plasmon resonance of GNR. All measurements were performed at room temperature using 10-mm cuvettes. X-ray photoelectron spectroscopy (XPS) measurements were conducted using an ESCA Laboratory Thermo Scientific Theta Probe spectrometer (Waltham, MA, USA) with monochromatic Al Kα radiation (1,486.68 eV). C (1*s*) peak was used as an internal standard calibration peak at 284.6 eV.

## Results and discussion

**Figure 1:**
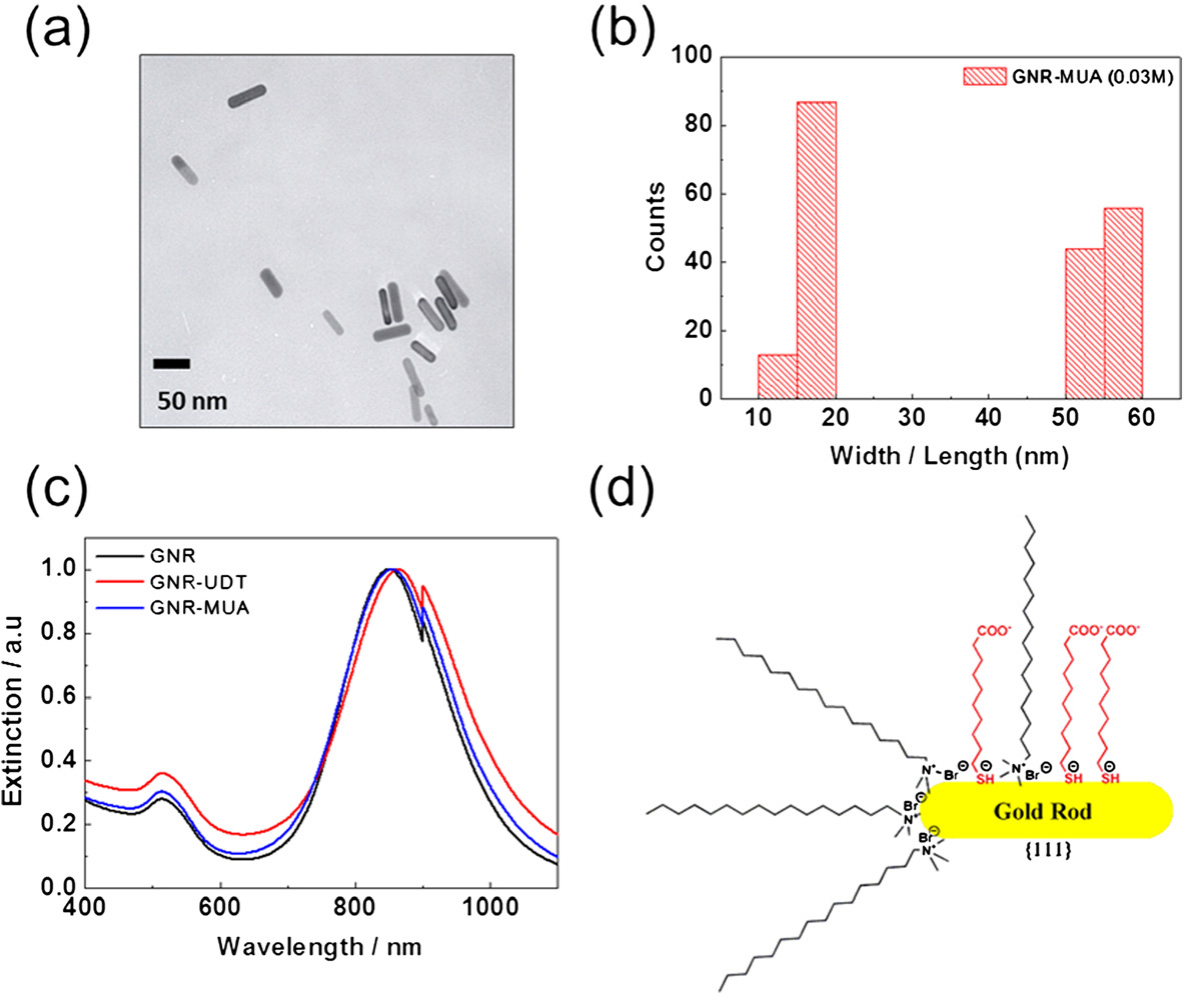
**TEM, size distribution, UV-visible-IR extinction spectra, and functionalized GNR ligand.** TEM images of GNR-MUA (**a**). Size distribution of GNR-MUA (**b**). Normalized UV-visible-IR extinction spectra of representative GNR-MUA in aqueous solutions (**c**). Schematic illustration of functionalized GNR ligand with CTAB, UDT, and MUA (**d**).

Figure 1a,b shows transmission electron microscopy (TEM) images and a particle size distribution of MUA which illustrates that no physical characteristic dissimilarity was found with as-synthesized GNR upon modification of GNR-MUA. The TEM image does not exhibit any corrosion, aggregation, or other defect (Figure 1a). The particle size analysis was carried out by counting about 100 particles for each specimen. It is estimated that the GNR has an average length of 53.93 ± 3.81 nm and diameter of 16.47 ± 1.76 nm, while the average length of as-synthesized GNR is 56.24 ± 3.47 nm and average diameter is 16.62 ± 1.60 nm (Figure 1b).

To ensure the integrity of each specimen and the formation of Au-S bond on GNR after MUA modification, we measured the characteristic extinction spectra, the XPS, and the zeta potential of as-synthesized GNR, GNR-MUA, and 1-undecanethiol modified gold nanorods (GNR-UDT) (Figure 1c). The LSPR spectral position is expected to be strongly affected by various factors such as the composition, formation and distribution of linkages, size, or shape of nanoparticles, as well as the refractive index of dielectric medium around them [26]. The as-synthesized GNR exhibited an absorption band centered at 850 nm. After the surface functionalization, a redshift of the extinction spectra was observed between GNR-MUA and GNR-UDT, at wavelengths 864 and 854 nm, respectively. The intensity of LSPR peak was found to be constant, but the FWHM of the peaks became broader for GNR-MUA and GNR-UDT as the gold-thiol bond formed [27].

XPS spectra measurement can confirm the formation of thiols bond to the Au surface. The XPS spectra shows that thiolates have S 2*p* binding energies of about 162.40 eV, whereas unbound thiols have those of 164 to 165 eV (Additional file 1: Figure S1). This result is identical with the results of Zhao et al. [28]. Here, the C 1*s* peak at 284.88 eV was used as an internal standard calibration peak. The results also indicated that MUA was successfully bound to the surface of GNR. We further certified the degree of this replacement through zeta potential of GNR-MUA (Table 1). GNR displayed a very positive zeta potential (58.08 ± 0.6 eV) when CTAB dispersed on the metal surface. It has been noticed that there was an apparently decrease of zeta potential GNR-MUA (29.4 ± 0.6 eV) when surface GNR was modified with MUA. Besides, as the pH of GNR-MUA was adjusted from acid to base condition, the zeta potential becomes almost neutral. This result supports that CTAB coverage of GNR is partially displaced (Table 1).

**Table 1:** Zeta potentials and pH of GNR, GNR-MUA, and GNR-MUA after adding 30 μL NaOH

	**Zeta potential**	**pH**
GNR	58.07 ± 0.55	3.92
GNR-MUA (0.03 M)	29.4 ± 0.6	7.49
GNR-MUA (+NaOH 30 μL)	8.69 ± 1.3	10.16

The face-selective modifications had been widely used in understanding and controlling the dynamics of self-assembled gold nanoparticles [29]. However, the mechanism of replacing CTAB is still an open question [30]. Here, the partially displaced surface can be explained by the following: First, according to the synthesis method of GNR by Sau et al., the GNR made in the presence of silver ions are single crystalline, with {111} facets on the long side of the rods [15]. On the other hand, it was reported that the surface energy of different facets generally increases in the order γ{111} < γ{100} < γ{110} [31]. Owing to the lowest surface energy among other facets and the particular structure of the {111} plane, thiol molecules like MUA and UDT preferentially bind onto it [32], which leads to the localization of monothiol MUA molecules prior at the long side of the rods (Figure 1d). Second, although the adsorption of a HS-containing aliphatic molecule onto the Au surface occurs very quickly, typically in few minutes at room temperature, Xia et al. believe that the presence of a compact bilayer of CTAB with high binding affinity to the surface of GNRs was responsible for the low coverage density of -S-PEG-NH_2_ chains on the CTAB-capped GNRs after ligand exchange [33].

**Figure 2:**
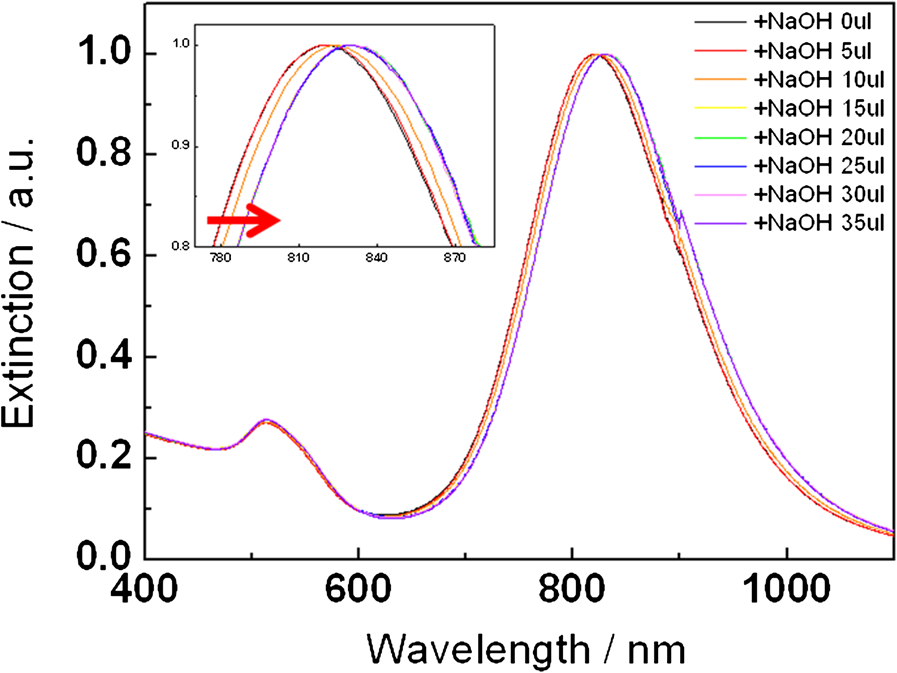
LSPR redshift of GNR-MUA after NaOH was added.

To gain more insight about the relationship between LSPR and pH value, the plasmonic effect on the GNR-tethered MUA as a function of pH was studied using acid–base titration methods [34]. As Figure 1 shows, a 10.5 nm of LSPR shift of GNR-MUA (821.5 to 832 nm) was found after 30 μL of NaOH was added, similar to the result of Zijlstra et al., in which approximately 8-nm shift was detected with biotin receptors when the binding of single protein occurs [21]. At the same time, the plasmon peak exhibits redshift with increasing pH (pH 6.41 to 8.88) (Figure 2). It is noteworthy that this peak shift is not due to the aggregation of GNR because the self-assembly of GNR would led to a decrease in the absorption of the long wavelength band, accompanied by the formation of a redshifted absorption band [29,35].

**Figure 3:**
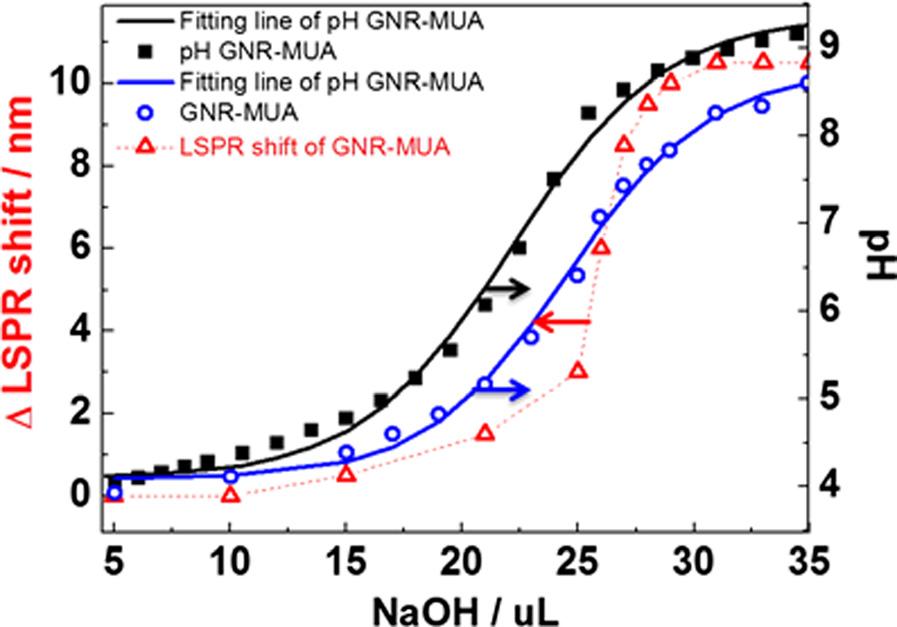
LSPR shift of GNR-MUA versus NaOH volume.

In addition, Figure 3 specifically summarizes the results of the absorption spectrum and the plasmon band intensity in a pH range of 3.8 to 8.88. It reveals a sigmoidal relation between LSPR shift and the volume of NaOH, when a 1- to 5-μL interval of NaOH was added. The sigmoidal curves of GNR-MUA (blue) before and after carboxylic acid deprotonation (red) seem to be right shifted compared with pure MUA (black) curve as a higher pKa value was found after MUA bound onto the metal surface [36]. Nevertheless, the position of LSPR band GNR-MUA added with different amounts of NaCl solutions (same concentration with NaOH) remain constant, which confirmed that the observed LSPR shift GNR-MUA was solely attributed to the pH changes instead of the combination effect from ionic strength (Additional file 1: Figure S2). According to Sethi et al., a dramatic broadening and shift in LSPR that are caused by electrostatic aggregation of GNRs can occur in solution based simply upon the anions of the solvent used [37]. The addition of an analyte will induce the aggregation of nanoparticles, and the plasmon band will redshift due to coupling of surface plasmon.

**Figure 4:**
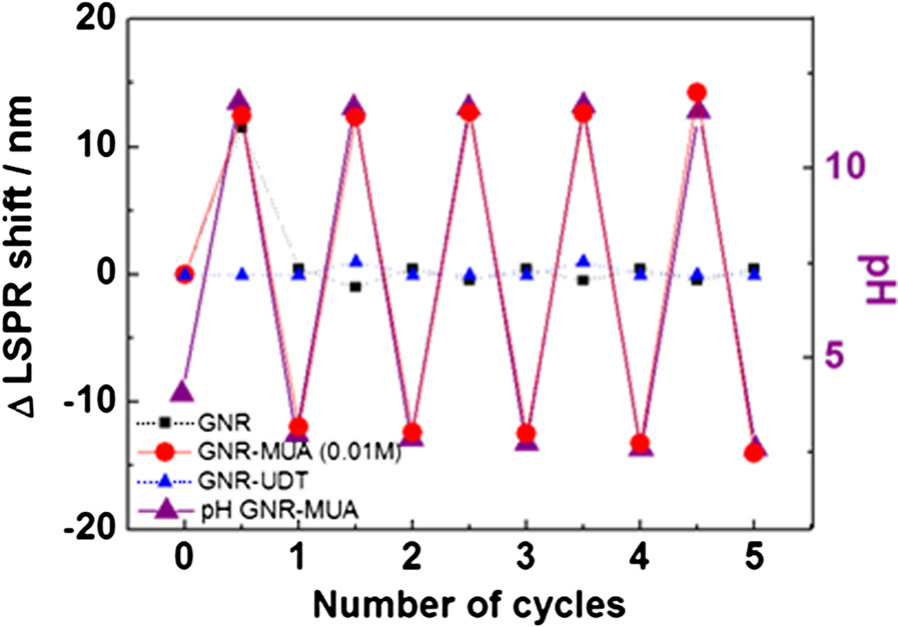
Reversibility of LSPR shift from GNP, GNP-UDT, and GNP-MUA between pH 2.60 and 11.75.

Simultaneously, to verify that the LSPR shift of GNR-MUA was related to the charge on the surface of GNR, both LSPR of as-synthesized GNR and GNR-UDT were also estimated in the pH range of 3.8 to 8.88 (Figure 4). GNR-UDT is used here as a control which has the same chain length with GNR-MUA but uncharged terminal group. However, no LSPR shift was found. Moreover, the zeta potential of GNR-MUA also exhibits the critical role of functional group charge towards LSPR shift that decreases from 29.4 ± 0.6 mV to 8.69 ± 1.3 mV after adding 30 μL NaOH (Table 1). Furthermore, to verify the influence of free MUA in the solution towards the LSPR shift, we found that there was a consistence LSPR shift trend between washed and unwashed GNR-MUA samples. These results demonstrated that the observation of pH-dependent LSPR shift was apparently related to the changes in the charge of the carboxylic acid groups of MUA bond on GNR instead of free carboxylic groups of MUA (Additional file 1: Figure S3).

Based on the above observation, subsequent experimental efforts have focused on the reversibility of the system. The titration procedure was repeated several times, going up and down on the pH scale. The LSPR of as-synthesized GNRs and GNR-UDT remains unchanged after the addition of 30 μL NaOH/HNO_3_ (Figure 4). This result is in good agreement with the result presented above that the LSPR of as-synthesized GNR and uncharged GNR-UDT was definitely not influenced by pH fluctuation. In comparison, the LSPR shift of GNR-MUA as a function of pH was found to be reversible between pH 11.75 and pH 2.60. Hence, these results indicate that the reversible change to the plasmon of these GNR tethered with MUA shows pH dependence, and this phenomenon demonstrates the utility of our pH nanosensor in a specific range of pH conditions.

**Figure 5:**
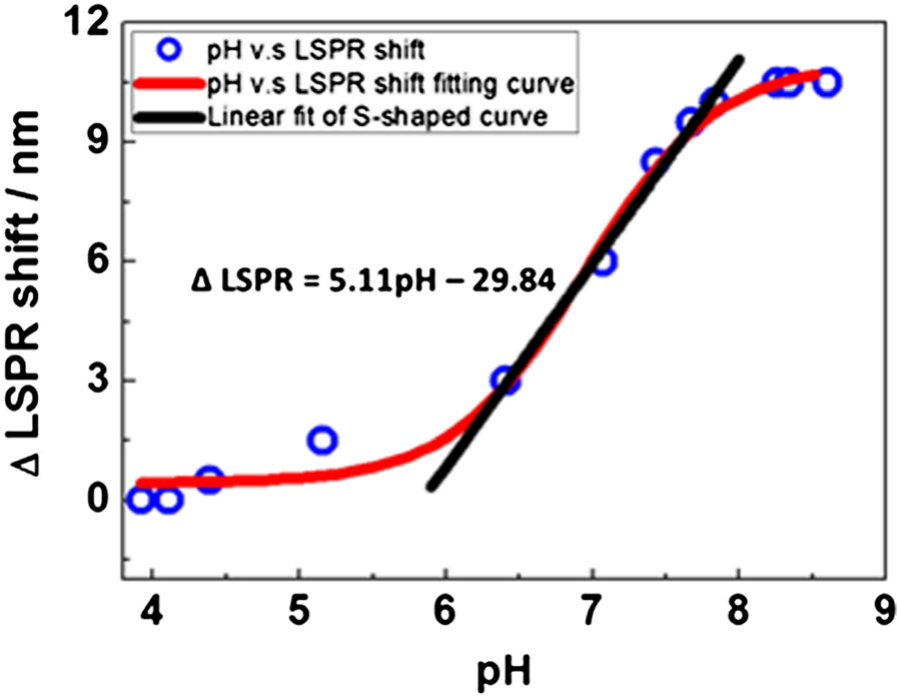
LSPR shift of GNR-MUA ligands as a function of pH in solution.

The LSPR shift of GNR-MUA is 10.5 nm (821.5 to 832 nm) within the pH range of 6.41 to 8.88 (Figure 5). The S-shaped curve has a linear response range between pH 6.41 and 7.83. The slope of 5.11 indicated that there was a 5-nm shift of LSPR for each unit change of pH value. This pH-sensing range suggests potential application for pH determination in living-cell organelles such as endosomes and lysosomes, especially for the detection of specific tumor cells for which the cellular pH is within a range between 6.40 and 6.90 [17].

**Figure 6:**
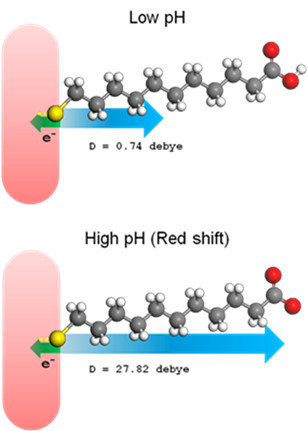
**Schematic of electron-pulling force.** On GNR-MUA to cause blue/red wavelength shift of LSPR at low and high pH.

It is well established that the peak wavelength, *λ*_max_, of the LSPR is dependent upon the size, shape, and distance between nanoparticles, as well as its dielectric properties and the changes in the effective refractive index (RI) of local surrounding environment including substrate, solvent, and adsorbates [38]. The dependence of LSPR or Fano resonance peak maximum [39] on RI which changes near the metal surface has been utilized in many plasmonic sensing applications. According to the modified equation of the LSPR wavelength shift Δ*λ*_max_ = *m*Δ*n*(*t*/*l*) by Malinsky et al. [40], where *m* is the refractive index sensitivity, Δ*n* represents the difference of local refractive index of protonated (*n*_COOH_) and deprotonated ( $n_{{\mathrm{COO}}^{-}}$) MUA, and *t* and *l* symbolize the thickness of the surrounding GNR after modification and decay length of the LSPR, individually in this experiment. From our refractive index measurements, there was no statistically significant difference between $n_{{\mathrm{COO}}^{-}}$ and *n*_COOH_. This suggests that there are very little changes in the local dielectric environment of protonated/deprotonated GNR-MUA nanoparticles. Therefore, our observation is not concordant with the equation mentioned above. However, the adsorption of thiol organic molecules can lead to the formation of microscopic surface dipoles that will modify the energy level alignment at the interface in both bulk and quantum dot semiconductors as observed in photovoltaic applications [41]. Here, the dipole moments calculated by DFT method for protonated and deprotonated MUA are 0.7 and 27.5 Debye, respectively (Figure 6). Thus, it is plausible that the redshift observed at higher pH is attributed to a relatively higher dipole moment of MUA as it is deprotonated. It is noteworthy that the formation of Au-thiol covalent bond shifts the LSPR to shorter wavelengths by approximately 10 nm, and it is due to the electron-donating nature of the sulfur headgroup in the molecule [42]. This means that the occurrence of the blueshift upon GNR happened while additional electrons were gained, while a redshift happened when part of the electrons were lost from the surface of GNR. The protonated/deprotonated MUA ligand that caused changes in the dipole moment of molecules may trigger various degrees of electron pulling force (the carboxyl groups of MUA are electron-withdrawing groups [43]). At a high pH, a larger electron-pulling force that restrains the electron-donating process of sulfur atom on MUA to the Au rod may cause the shift of LSPR to longer wavelengths, while a relative blueshift of LSPR occurs for GNR-MUA for a lower pH (Figure 6).

## Conclusions

In conclusion, a pH-dependent wavelength shift has been observed in GNR-MUA, which suggests that the charges formed on the surface of GNR after protonation/deprotonation of the carboxylic ligands of MUA play an important role by modulating LSPR phenomenon around the functionalized gold nanorods. Otherwise, -CH_3_-terminated ligand (CTAB or MUA) is independent of pH. The free MUA in the solution will not affect the LSPR shifting. In addition, we confirmed that the LSPR shifting is neither aggregation-induced optical signal nor the change of ionic strength. The LSPR shift of GNR is attributed to the dipole moment change after protonation/deprotonation of carboxylic groups of MUA. This GNR-MUA-based sensor can offer a 5-nm shift of LSPR for a unit change of pH value. Although the sensitivity of this GNR-MUA still has room for further improvement, such a stable and easily prepared GNR-MUA has potential to become efficient and promising pH nanosensors to study intra- or extra-cellular pH in a wide range of chemical or biological systems.

## Competing interests

The authors declare that they have no competing interests.

## Authors’ contributions

PY conceived and designed all the experiments. Y-RT performed all the experiments and wrote the manuscript. XW, JT, and TH participated in the discussion. All authors read and approved the final manuscript.

## Supplementary Material

Additional file 1: Figures S1 to S3 **Figure S1.** X-ray photoelectron spectroscopy (XPS) high-resolution spectra of C (1*s*) and S (2*p*) for MUA (a and b). **Figure S2.** (a) UV-visible-IR extinction spectra of representative GNR-MUA added with NaCl. (b) The dependence of the LSPR shift upon the concentration of NaCl. **Figure S3.** Reversibility of LSPR shift from unwashed GNR-MUA between pH 6.31 and 10.65. Click here for file
