# Supplementary material for: Induced pH-dependent shift by local surface plasmon resonance in functionalized gold nanorods
Source: Nanoscale Res Lett. 2013 Feb 22;8(1):103. doi: 10.1186/1556-276X-8-103 (PMC3599965; doi:10.1186/1556-276X-8-103)
Supplement: Additional file 1: Figures S1 to S3 — Figure S1. X-ray photoelectron spectroscopy (XPS) high-resolution spectra of C (1s) and S (2p) for MUA (a and b). Figure S2. (a) UV-visible-IR extinction spectra of representative GNR-MUA added with NaCl. (b) The dependence of the LSPR shift upon the concentration of NaCl. Figure S3. Reversibility of LSPR shift from unwashed GNR-MUA between pH 6.31 and 10.65. [file 1556-276X-8-103-S1.doc]

Supplementary Material (ESI) for *Nanoscale Research Letters*

**Supplementary Information**

**Induced pH-Dependent Shift by Local Surface Plasmon Resonance in Functionalized Gold Nanorods**

Yon-Rui Toh^1^, Pyng Yu^1^, Xiaoming Wen^1^, Jau Tang^1^* and T. S. Hsieh^2^

^1^Research Center for Applied Sciences, Academia Sinica, Taiwan

^2^Institute of Cellular and Organismic Biology, Academia Sinica, Taiwan

In the following, we provide three supporting illustrations which are mentioned in our man text.


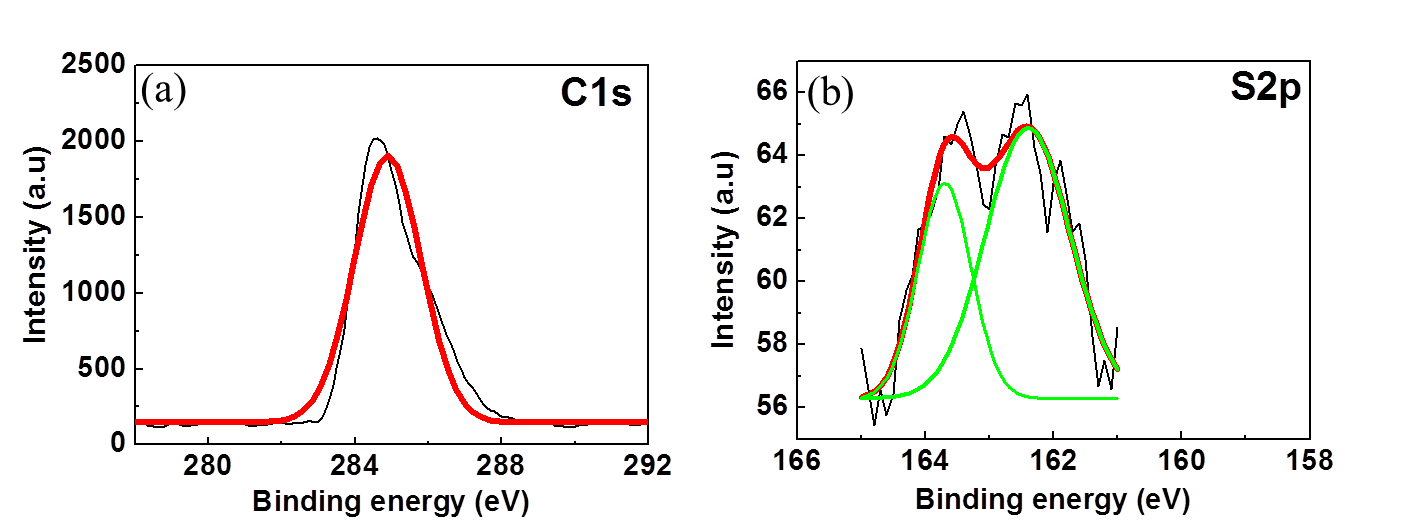


Figure S1. X-ray photoelectron spectroscopy (XPS) high-resolution spectra of C(1s) and S(2p) for MUA (a and b).


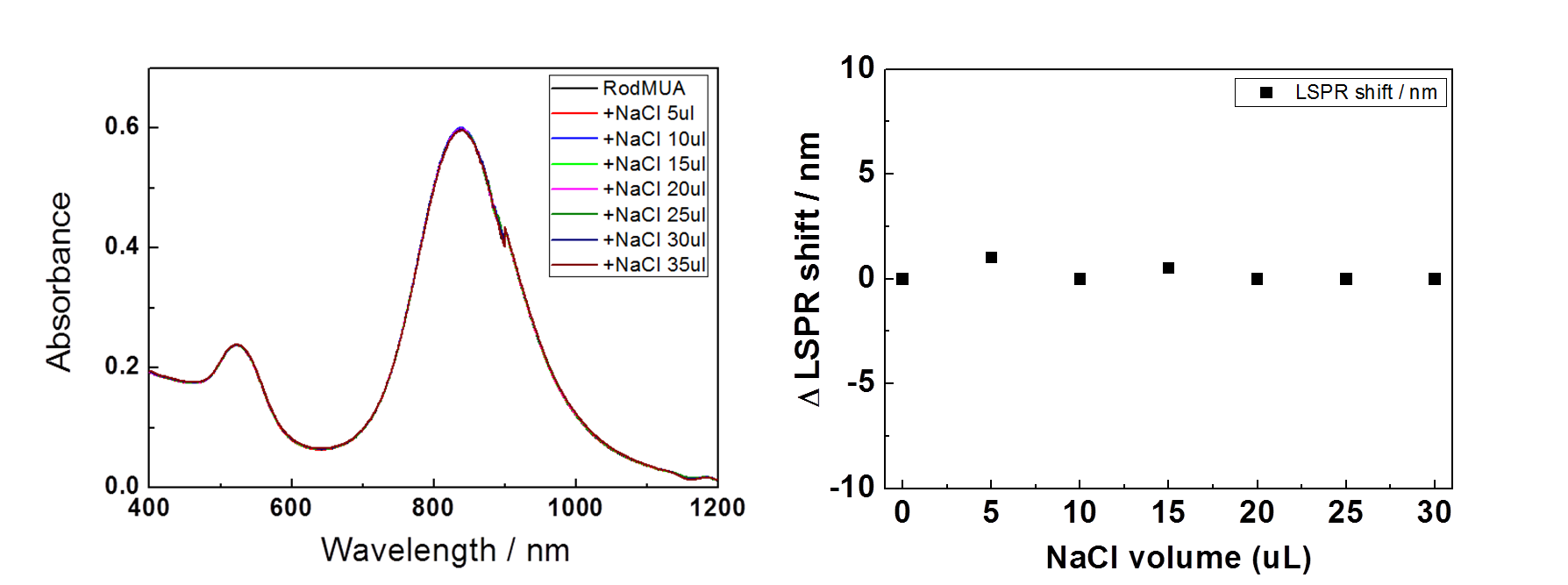


Figure S2 (a) UV-visible-IR extinction spectra of representative GNR-MUA added with NaCl. (b) The dependence of the LSPR shift upon the concentration of NaCl.


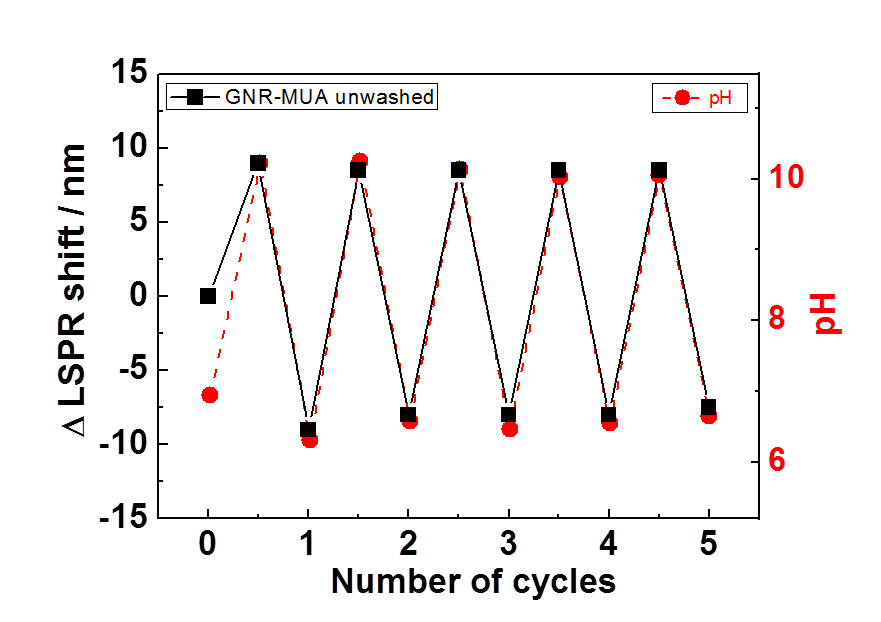


Figure S3. Reversibility of LSPR shift from unwashed GNR-MUA between pH 6.31-10.65.
